# Point Prevalence and Associated Factors of Hip Displacement in Pediatric Patients With Mitochondrial Disease

**DOI:** 10.3389/fped.2021.637240

**Published:** 2021-11-04

**Authors:** Sungmin Kim, Young-Mock Lee, Kun-Bo Park, Minsu Lee, Hoon Park

**Affiliations:** ^1^Department of Orthopedic Surgery, Chonnam National University Medical School and Hospital, Gwangju, South Korea; ^2^Department of Pediatrics, Gangnam Severance Hospital, Yonsei University College of Medicine, Seoul, South Korea; ^3^Division of Pediatric Orthopaedic Surgery, Severance Children's Hospital, Yonsei University College of Medicine, Seoul, South Korea; ^4^Department of Orthopaedic Surgery, Gangnam Severance Hospital, Yonsei University College of Medicine, Seoul, South Korea

**Keywords:** mitochondrial disease, mitochondrial myopathy, Leigh syndrome, MELAS syndrome, hip displacement

## Abstract

**Objective:** Mitochondrial disease is a multisystem disorder resulting from mitochondrial dysfunction. Although musculoskeletal system is vulnerable to mitochondrial dysfunction, little information is available on orthopedic issues such as hip displacement and scoliosis in patients with mitochondrial disease. We aimed to examine the point prevalence of hip displacement and investigate the associated factors in patients with mitochondrial disease.

**Methods:** We retrospectively reviewed the medical records and plain radiographs of patients diagnosed with mitochondrial disease between January 2006 and January 2019 at a single institution. Data, including patient age, sex, follow-up duration, syndromic diagnosis, and gross motor function were collected. Migration percentage was measured on the radiographs. The clinical and radiologic variables were compared between patients classified according to the presence of hip displacement and motor function level.

**Results:** We included 225 patients (135 men, 90 women). The mean age at the latest follow-up was 11.1 years, and the mean follow-up duration was 7.0 years. Hip displacement was noted in 70 (31.1%) patients. The proportion of patients with Leigh disease (*p* = 0.007) and the ratio of non-ambulators (*p* < 0.001) were higher among patients with hip displacement. The proportion of patients with Leigh disease was higher in the non-ambulators than the ambulators.

**Conclusion:** One-third of patients with mitochondrial disease developed hip displacement. Hip displacement was more common in non-ambulators or patients with hypertonia. Careful and serial monitoring for hip problems is required for non-ambulatory patients with mitochondrial disease who have increased muscle tone.

## Introduction

Mitochondrial disease is a complex and heterogenous multisystem disorder resulting from mitochondrial dysfunction and, particularly, from defects of the mitochondrial respiratory chain (MRC) and associated abnormal oxidative phosphorylation (1). Different phenotypes and genotypes of mitochondrial disease may be noted in patients with abnormality in mitochondrial DNA or nuclear DNA, with variations in the extent and severity of the manifestations owing to the defects in any of the numerous mitochondrial pathways (2, 3). Clinical symptoms may develop at any age and may affect any organ (1).

The musculoskeletal system consists of organs with high-energy requirements and is vulnerable to mitochondrial dysfunction (1). Orthopedic manifestations are noted in patients with mitochondrial disease from a variety of causes, including underlying myopathy, neuropathy, and abnormalities of tone (4). Musculoskeletal involvement is noted in progressive proximal myopathy, exercise intolerance, muscle weakness, and gait disturbance (3, 5, 6). Some patients with mitochondrial disease show severe musculoskeletal features, such as muscular contractures, scoliosis, hip dislocation, and limb deformities which may require surgical intervention. Only a few case reports on neuromuscular scoliosis in patients with mitochondrial disease have been published to date (7, 8).

Hip displacement can result in pain and difficulty during sitting and perineal care, leading to a considerable negative impact on the quality of life (9). Although many studies have reported on hip displacement in patients with neuromuscular diseases, like cerebral palsy, muscular dystrophy, or spinal muscular atrophy (9–11), to the best our knowledge, the occurrence and related factors of hip displacement in patients with mitochondrial disease have not been reported previously. This study aimed to examine the frequency of hip displacement in patients with mitochondrial disease and investigate the factors associated with the occurrence of hip displacement in these patients.

## Materials and Methods

We reviewed the medical records of patients who were diagnosed with mitochondrial disease and regularly followed up at our hospital between January 2006 and January 2019 at a single institution. Mitochondrial disease was diagnosed according to the modified criteria for mitochondrial disease proposed by Bernier et al. (12), which include clinical, histopathologic, enzymatic, and metabolic parameters. The inclusion criteria for the present study were as follows: availability of plain radiographs of the hip and spine for the evaluation and proper clinical records for assessing the medical and ambulatory status.

Data, including patient age at the initial and latest follow-up, sex, follow-up duration, gross motor function, and muscle tone were collected. The age of the patients when hip displacement occurred was estimated using serial radiographs. Gross motor function was assessed according to the walking status. Patients were divided into three groups—independent ambulators, ambulators with an assistive device, and non-ambulators. Muscle tone was classified as hypertonia, hypotonia, and normal. We investigated whether these patients underwent surgery. The type of surgery and postoperative complications were noted.

All patients were classified according to the syndromic diagnosis. The diagnoses of mitochondrial encephalomyopathy, lactic acidosis, and stroke-like episodes (MELAS) and Leigh disease were confirmed based on the diagnostic criteria reported by Yatsuga et al. (3) and Rahman et al. (13), respectively. Patients with non-specific and non-categorized mitochondrial disease did not show typical clinical symptoms, abnormal biochemical results, or genetic mutations conforming to the known and established mitochondrial syndromes (14). Genetic analysis and spectrophotometric biochemical enzyme assay for MRC complex of the muscle were performed for all patients.

Anteroposterior pelvic radiographs, taken with the hips slightly internally rotated, were used for radiologic measurements. All measurements were performed by two orthopedic surgeons. The migration percentage (15) and pelvic obliquity (16) were measured on pelvic radiographs at the initial and last follow-up. Hip subluxation requiring reconstructive surgery was defined according to the current literature as migration percentage >30%, and hip dislocation was defined as migration percentage of 100% (17, 18). The degree of pelvic obliquity, the angle made by the horizontal line and line between the lowest points of the pelvic bones on the right and left sides, was also checked. The Cobb angle was measured from anteroposterior whole-spine radiographs. Scoliosis was defined as Cobb angle >10°.

All data were analyzed using SPSS version 23 software (IBM Corp., Armonk, NY, USA). To compare the variables between groups with and without hip displacement, the two-sample *t*-test or Mann-Whitney *U*-test was used for continuous variables, and the χ^2^-test or Fisher's exact test was used for categorical variables. *p*-value < 0.05 was considered significant for all analyses.

## Results

We identified 258 patients with mitochondrial disease. Thirty-three patients were excluded owing to inadequate radiographs for hip evaluation. The remaining 225 patients (135 men and 90 women) were included in this study. According to the syndromic diagnosis, 81 patients had Leigh syndrome, 10 had MELAS, and 134 had non-specific mitochondrial disease. Among 81 patients who met the clinical criteria for Leigh syndrome, 16 patients had confirmed the mitochondrial DNA mutation. Two patients showed nuclear DNA mutation and had *SURF1* gene mutation. Ten patients diagnosed with MELAS were all positive mitochondrial DNA 3243A>G mutation. The majority of patients were not genetically confirmed in this study. The mean age at the latest follow-up was 11.1 ± 5.3 years, and the mean age at the initial visit was 4.8 ± 3.9 years. The mean follow-up duration was 7.0 ± 3.9 years (range, 1–24 years).

**Figure 1:**
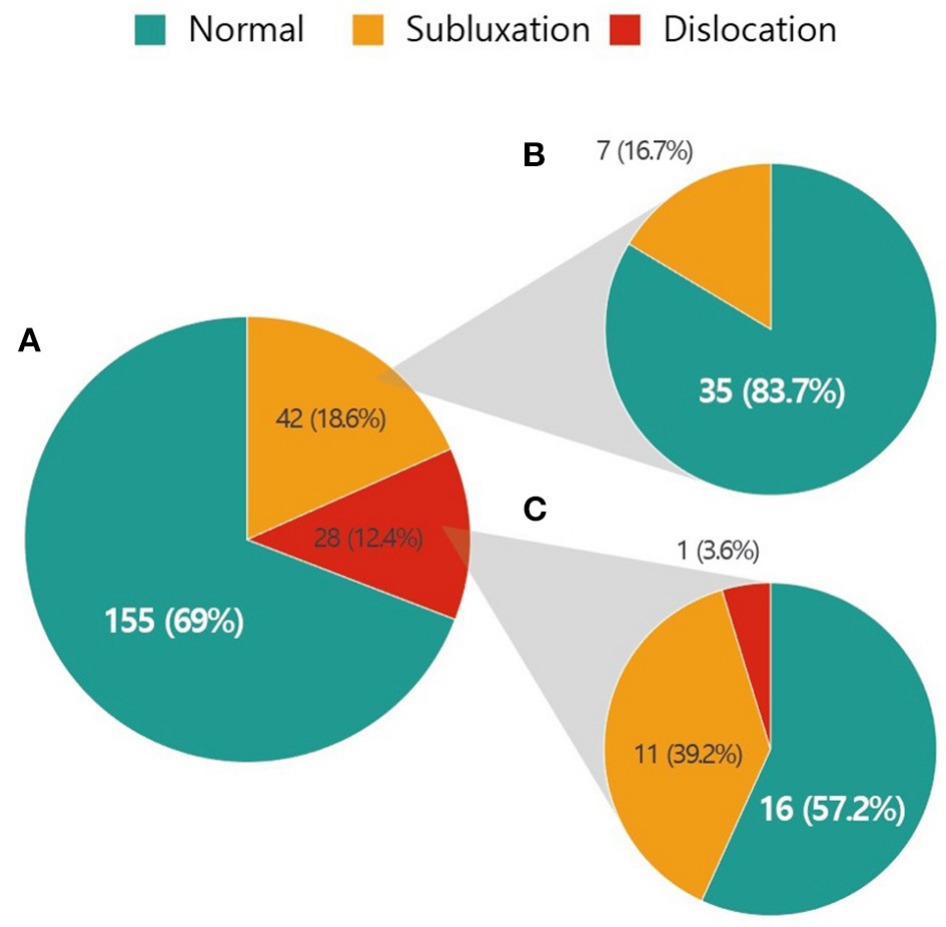
Incidence of hip subluxation and dislocation in patients with mitochondrial disease and status of the hip at the initial assessment. **(A)** Number of patients with hip subluxation and dislocation. **(B)** Initial status of the hip in patients with hip subluxation. **(C)** Initial status of the hip in patients with hip dislocation.

Hip displacement was noted in 70 (31.1%) patients. The estimated mean age when hip displacement occurred was 7.9 (3.2–12.7) years. The proportion of patients with hip subluxation and hip dislocation was 18.7 and 12.4%, respectively (Figure 1A). The status of the hip at the initial radiographic examination in patients with hip subluxation and dislocation is illustrated in Figures 1B,C, respectively. At the first assessment, 35 (83.3%) of the 42 patients with hip subluxation were normal and 16 (57.1%) of the 28 patients with hip dislocation were normal. According to syndromic diagnosis, the frequency of hip displacement was 43.2% (35 of 81) in patients with Leigh syndrome, 10% (1 of 10) in patients with MELAS, and 25.4% (34 of 134) in patients with non-specific and non-categorized mitochondrial disease.

**Figure 2:**
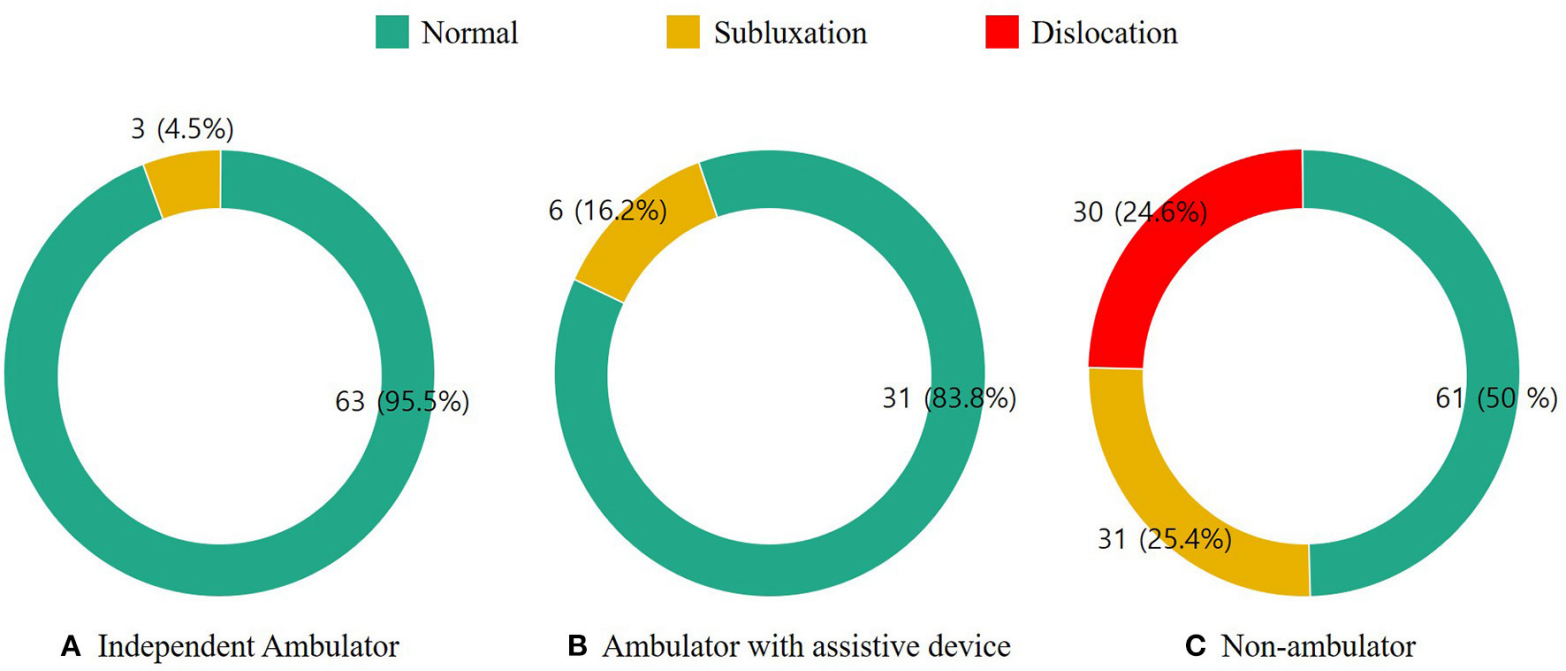
Hip displacement in patients with mitochondrial disease according to the motor function level. **(A)** Independent ambulatory, **(B)** ambulator with assistive device, and **(C)** non-ambulator.

Sixty-six (29.3%) patients were independent ambulators, 37 (16.4%) were able to ambulate with an assistive device, and 122 (54.2%) were non-ambulators. The distribution of hip displacement according to motor function level is presented in Figure 2. The occurrence of hip displacement was found in 9 (8.7%) patients who were able to walk and in 61 (50%) non-ambulatory patients. The frequency of hip subluxation was 4.5% in independent ambulators, 16.2% in ambulators with an assistive device, and 25.4% in non-ambulators. While hip dislocation was not found in ambulatory patients, it was noted in 30 (24.6%) non-ambulatory patients.

Seventy-five (33.3%) patients had increased muscle tone, 29 (12.9%) patients had decreased muscle tone, and 72 (32%) patients showed normal muscle tone. The muscle tone in 49 (21.8%) patients cannot be evaluated because of the lack of medical record. Hypertonia was found in 39 (48.1%) patients with Leigh syndrome, and 35 (26.1%) patients with non-specific mitochondrial disease.

Mitochondrial respiratory chain complex deficiency was found in 191 (84.8%) patients, and the results showed complex I deficiency in 165 cases (86.4%), complex II deficiency in one case (0.5%), and complex IV deficiency in 25 cases (13.1%).

Forty-three (19.1%) patients had scoliosis, and the mean Cobb's angle was 48.2°. Hip displacement was noted in 16 patients with scoliosis. Pelvic obliquity was present in 7.1%, and the mean pelvic obliquity was 19.3°.

Comparisons of the characteristics between patients with and without hip displacement are described in Table 1. The proportion of patients with Leigh disease was higher among patients with hip displacement than among those without (*p* = 0.007). Non-ambulators were more commonly noted among the group with hip displacement (*p* < 0.001). There was a significant difference in the mean follow-up period between the two groups. Significant differences in age, sex, MRC deficiency, and presence of scoliosis and pelvic obliquity were not found between the two groups.

**Table 1:** Distribution of the variables according to hip displacement.

	**Hip displacement (*n* = 70)**	**No hip displacement (*n* = 155)**	***p*-Value**
Age, mean ± SD, years	10.4 ± 3.5	11.6 ± 6.4	0.076
Sex, *n* (%)			0.661
Male	40 (57.1)	94 (60.6)	
Female	30 (42.9)	61 (39.4)	
Mean follow-up, mean ± SD, years	8.4 ± 3.3	6.7 ± 3.9	0.021
Syndrome, *n* (%)			0.007
Leigh	35 (50.0)	46 (29.7)	
MELAS	1 (1.4)	9 (5.8)	
NS&NC MD	34 (48.6)	100 (64.5)	
Motor function level, *n* (%)			<0.001
Ambulatory			
Without aids	3 (4.3)	63 (40.6)	
With aids	6 (8.6)	31 (20.0)	
Non-ambulatory	61 (87.1)	61 (39.4)	
Muscle tone, *n* (%)			<0.001
Hypertonia	42 (60.0)	33 (21.3)	
Hypotonia	12 (17.1)	17 (11.0)	
Normal tone	5 (7.1)	67 (43.2)	
Unknown	11 (15.7)	38 (24.5)	
MRC deficiency, *n* (%)			0.174
Complex I deficiency	47 (67.1)	118 (76.1)	
Complex II deficiency	1 (1.4)	0 (0)	
Complex IV deficiency	11 (15.7)	14 (9.0)	
Absence of MRC deficiency	11 (15.7)	23 (14.8)	
Scoliosis, *n* (%)			0.362
Yes	16 (22.9)	27 (17.4)	
No	54 (77.1)	128 (82.6)	
Pelvic obliquity, *n* (%)			0.100
Yes	8 (11.4)	8 (5.2)	
No	62 (88.6)	147 (94.8)	

*MELAS, mitochondrial encephalomyopathy, lactic acidosis, and stroke-like episodes; NS&NC MD, non-specific and non-categorized mitochondrial disease; MRC, mitochondrial respiratoy chain complex*.

We also compared the variables between ambulators and non-ambulators (Table 2). Age (*p* = 0.012), distribution of syndromic diagnosis (*p* = 0.027), and presence of scoliosis (*p* = 0.027) significantly differed between the two groups. The proportions of patients with Leigh disease and scoliosis were higher among the non-ambulators.

**Table 2:** Variables distribution according to ambulation.

	**Ambulatory**	**Non-ambulatory**	***p*-Value**
	**(*n* = 103)**	**(*n* = 122)**	
Age, mean ± SD, years	12.3 ± 5.8	10.4 ± 5.4	0.012
Sex, *n* (%)			0.222
Male	66 (64.1)	68 (55.7)	
Female	37 (35.9)	54 (44.3)	
Syndrome, *n* (%)			0.027
Leigh	28 (27.2)	53 (43.4)	
MELAS	4 (3.9)	6 (4.9)	
NS&NC MD	71 (68.9)	63 (51.7)	
Muscle tone, *n* (%)			<0.001
Hypertonia	19 (18.4)	56 (45.9)	
Hypotonia	3 (2.9)	26 (21.3)	
Normal tone	58 (56.3)	14 (11.5)	
Unknown	23 (22.3)	26 (21.3)	
Scoliosis, *n* (%)			0.027
Yes	13 (12.6)	30 (24.6)	
No	90 (87.4)	92 (75.4)	
Pelvic obliquity, *n* (%)			0.118
Yes	4 (3.9)	12 (9.8)	
No	99 (96.1)	110 (90.2)	

*MELAS, Mitochondrial encephalomyopathy, lactic acidosis, and stroke-like episodes; NS&NC MD, non-specific and non-categorized mitochondrial disease*.

Ten patients underwent surgical treatment for hip displacement. Among five patients with Leigh syndrome, three patients underwent hip reconstruction surgery (femur and pelvic osteotomy) while other two underwent soft tissue release. Hip reconstruction surgery was done in five patients with non-specific mitochondrial disease. There was no redislocation of hip in patients managed with surgery during the study period. Three patients received the posterior spinal fusion for scoliosis. Bracing was used in 10 patients, and wheelchair modification, physical therapy, and environmental adaptation were used in the remaining 30 patients. All patients who underwent surgery had increased muscle tone.

## Discussion

This is the first report to describe the occurrence of hip displacement in patients with mitochondrial disease. Hip displacement developed in 31.1% of patients with mitochondrial disease in our cohort. The point prevalence of hip displacement in our study is similar to that reported in patients with cerebral palsy (18, 19). The true incidence of hip displacement in patients with mitochondrial disease is difficult to determine because of the rarity of mitochondrial disease. However, the number of patients in our cohort is large enough to be considered meaningful.

In our study, the status of the hip in most patients with hip displacement was normal at the initial visit. This may have been because of either late-onset hip displacement or the failure to detect hip displacement at the initial assessment. Although it is unclear whether hip displacement is present at birth or can develop over time, our results imply that hip displacement in patients with mitochondrial disease may progress with age. This result suggests that hip displacement should be regularly monitored in patients with mitochondrial disease.

Our results showed that hip displacement was strongly associated with the motor function level. The frequency of hip displacement was higher in non-ambulators. These results were similar to those of other studies that confirmed the linear association between Gross Motor Function Classification System (GMFCS) level and hip displacement in patients with cerebral palsy (18, 19). As the GMFCS is specific to cerebral palsy and cannot be directly applied to patients with mitochondrial disease, we classified the patients in this study into three groups according to motor function level, as follows: independent ambulators, ambulators with an assistive device, and non-ambulators. Dislocation was not noted in ambulatory patients and was noted only in non-ambulatory patients. The present data showed that non-ambulatory patients with mitochondrial disease had a high risk for hip displacement or dislocation. Therefore, physicians must pay more attention to non-ambulatory patients with mitochondrial disease.

Our study reported that the proportion of patients with Leigh disease was higher in the group with hip displacement. Leigh syndrome is a neurodegenerative disease characterized by bilateral central nervous system lesions (14). Leigh syndrome is a heterogenous neurodegenerative condition that affects the basal ganglia, the thalamus, and the brainstem (20). Affected individuals may develop weak muscle tone (hypotonia), involuntary muscle contractions (dystonia), muscle spasm (spasticity), and problems with movement and balance (ataxia) (21). Although we could not identify the muscle tone in all included patients, the proportion of patients with hypertonia in Leigh syndrome was higher than that of non-specific mitochondrial disease. This result may support that increased muscle tone is the main factor causing the hip displacement in patients with Leigh syndrome. Although spasticity is an important cause of hypertonia, increased muscle tone may also be attributable to dystonia when there is simultaneous co-contraction of agonist and antagonist muscles at rest (22). It is well-known that dystonia is one of common feature of mitochondrial disease such as Leigh syndrome (21). Muscle imbalance due to increased or spastic muscle tone induce abnormal force on the hip joint (18, 23). Over time, the relatively increased activity of the hip adductors and flexors abnormally alter the joint reactive force, gradually causing the proximal femur to subluxate from the acetabulum (17). These forces also create anatomic differences and prevent the physiologic remodeling of immature hip geometry including increased anteversion, increased neck-shaft angle, and acetabular dysplasia (24). The resulting pathology can lead to hip subluxation and eventual dislocation, which may deteriorate the motor function level. Although, muscular imbalance can also occur in patients with hypotonic or flaccid muscle tone, hip displacement is common in patients with increased or spastic muscle tone such as in cerebral palsy. We assume that hypertonia is the main factor causing hip displacement even in patients with mitochondrial disease.

The frequency of scoliosis in patients with mitochondrial disease was 19.1%. Only two case reports on scoliosis in patients with mitochondrial disease have been published to date (7, 8). Although they reported a 5% incidence of scoliosis among patients with mitochondrial myopathies, the total number of patients was only 60. Therefore, it is not reasonable to compare this rate with that in our study.

The distribution of scoliosis according to the presence of hip displacement was not different. Scoliosis is frequently observed in patients with neuromuscular disease (14, 25–27). Although hip displacement may accompany scoliosis, previous studies investigating the causal relationship between these factors showed inconsistent results (28–33). Further studies are required to evaluate the association between hip displacement and scoliosis in patients with neuromuscular diseases.

The proportion of patients with scoliosis was higher among the non-ambulators than among the ambulators. In patients with cerebral palsy, the risk of scoliosis was closely associated with the ambulation status (34). Low motor function level due to muscle weakness and incomplete muscle control contributes to impaired trunk control and the development of spinal deformity. Our results showed that ambulation status was related to preserve the development of the hip joint in patients with mitochondrial disease.

Although we found a high incidence of hip displacement in patients with mitochondrial disease, there are no specific treatment guidelines and surgical techniques for these patients. Some patients with mitochondrial disease have very unhealthy medical condition and show high mortality rates from sepsis or pneumonia (1). It may be unnecessary to evaluate and monitor the status of the hip joint in these patients. However, we consider that serial evaluation for hip displacement is important because it may be cost-effective to preserve the hip joint by physical therapy or minor surgery, like soft tissue release (6). Considering the estimated mean age when hip displacement occurred, we recommend that hip radiography examination, such as the cerebral palsy surveillance program, ideally be initiated by 6–8 years of age in non-ambulatory patients with mitochondrial diseases. Surveillance frequency is based on the child's age, GMFCS level, and muscle tone.

This study had some limitations. First, our study is retrospective and cross-sectional in nature. We could not examine the same periods in all patients owing to the variable periods of follow-up, which is possibly why we found a significant difference in the mean follow-up periods between patients with and without hip displacement. This may be because non-ambulators with hip displacement frequently visit the hospital for medical care or checkups for the hip joint status, whereas ambulators without hip displacement do not need additional radiography examination. Additionally, some patients had not reached skeletal maturity at the final assessment, which may have led to a bias in assessing the incidence of hip displacement. As noted earlier, this is the first report on hip displacement in a large patient group with mitochondrial disease. Second, we included a smaller proportion of patients with MELAS because it is an extremely rare disease subtype. Thus, the frequency of hip displacement in patients with MELAS must be interpreted with caution.

In conclusion, 31.1% of patients with mitochondrial disease developed hip displacement. Hip displacement was more common in non-ambulators or patients with increased muscle tone. Careful and serial monitoring for hip problems are recommended in non-ambulatory patients with mitochondrial disease who have increased muscle tone.

## Data Availability Statement

The original contributions presented in the study are included in the article/supplementary material, further inquiries can be directed to the corresponding author/s.

## Ethics Statement

The studies involving human participants were reviewed and approved by the Institutional Review Board of the Gangnam Severance Hospital, Seoul, Korea. Written informed consent for participation was not provided by the participants' legal guardians/next of kin because: Our research involved no more than a minimal risk to our subjects and we used the existing medical records and the radiographs.

## Author Contributions

The initial concept was prepared by Y-ML and HP. The initial draft was prepared by SK. Data was collected by ML and SK. Data analysis was performed by SK and K-BP. The final draft was prepared by SK, Y-ML, and HP. Intellectual contents were provided by K-BP and HP. All authors read the final manuscript and approved it for submission.

## Funding

This research was funded by a faculty research grant of Yonsei University College of Medicine, grant number 6-2020-0123. The APC was funded by Yonsei University College of Medicine.

## Conflict of Interest

The authors declare that the research was conducted in the absence of any commercial or financial relationships that could be construed as a potential conflict of interest.

## Publisher's Note

All claims expressed in this article are solely those of the authors and do not necessarily represent those of their affiliated organizations, or those of the publisher, the editors and the reviewers. Any product that may be evaluated in this article, or claim that may be made by its manufacturer, is not guaranteed or endorsed by the publisher.

## References

[1] Eom S Lee HN Lee S Kang HC Lee JS Kim HD. Cause of death in children with mitochondrial diseases. Pediatr Neurol. (2017) 66:82–8. 10.1016/j.pediatrneurol.2016.10.006 27843091

[2] Eom S Lee Y-M. Preliminary study of neurodevelopmental outcomes and parenting stress in pediatric mitochondrial disease Pediatr Neurol. (2017) 71:43.e1–9.e1. 10.1016/j.pediatrneurol.2017.01.019 28476522

[3] Yatsuga S Povalko N Nishioka J Katayama K Kakimoto N Matsuishi T. MELAS: a nationwide prospective cohort study of 96 patients in Japan. Biochim Biophys Acta. (2012) 1820:619–24. 10.1016/j.bbagen.2011.03.015 21443929

[4] Parikh S Goldstein A Karaa A Koenig MK Anselm I Brunel-Guitton C. Patient care standards for primary mitochondrial disease: a consensus statement from the Mitochondrial Medicine Society. Genet Med. (2017) 19:10.1038/gim.2017.107. 10.1038/gim.2017.107 28749475 PMC7804217

[5] McFarland R Taylor RW Turnbull DM. The neurology of mitochondrial DNA disease. Lancet Neurol. (2002) 1:343–51. 10.1016/S1474-4422(02)00159-X 12849395

[6] Kisler JE Whittaker RG McFarland R. Mitochondrial diseases in childhood: a clinical approach to investigation and management. Dev Med Child Neurol. (2010) 52:422–33. 10.1111/j.1469-8749.2009.03605.x 20163433

[7] Li Z Shen J Liang J. Scoliosis in mitochondrial myopathy: case report and review of the literature. Medicine (Baltimore). (2015) 94:e513. 10.1097/MD.0000000000000513 25674747 PMC4602742

[8] Hiniker A Wong LJ Berven S Truong CK Adesina AM Margeta M. Axial mitochondrial myopathy in a patient with rapidly progressive adult-onset scoliosis. Acta Neuropathol Commun. (2014) 2:137. 10.1186/s40478-014-0137-3 25223649 PMC4180433

[9] Huser A Mo M Hosseinzadeh P. Hip surveillance in children with cerebral palsy. Orthop Clin North Am. (2018) 49:181–90. 10.1016/j.ocl.2017.11.006 29499819

[10] Chan KG Galasko CS Delaney C. Hip subluxation and dislocation in Duchenne muscular dystrophy. J Pediatr Orthop B. (2001) 10:219–25. 10.1097/01202412-200107000-00012 11497366

[11] Sporer SM Smith BG. Hip dislocation in patients with spinal muscular atrophy. J Pediatr Orthop. (2003) 23:10–4. 10.1097/01241398-200301000-00002 12499935

[12] Bernier FP Boneh A Dennett X Chow CW Cleary MA Thorburn DR. Diagnostic criteria for respiratory chain disorders in adults and children. Neurology. (2002) 59:1406–11. 10.1212/01.WNL.0000033795.17156.00 12427892

[13] Rahman S Blok RB Dahl HH Danks DM Kirby DM Chow CW. Leigh syndrome: clinical features and biochemical and DNA abnormalities. Ann Neurol. (1996) 39:343–51. 10.1002/ana.410390311 8602753

[14] Carroll NC. Assessment and management of the lower extremity in myelodysplasia. Orthop Clin North Am. (1987) 18:709–24. 10.1016/S0030-5898(20)30360-6 3313170

[15] Reimers J. The stability of the hip in children: a radiological study of the results of muscle surgery in cerebral palsy. Acta Orthop Scand Suppl. (1980) 184:1–100. 10.3109/ort.1980.51.suppl-184.01 6930145

[16] Hagglund G Goldring M Hermanson M Rodby-Bousquet E. Pelvic obliquity and measurement of hip displacement in children with cerebral palsy. Acta Orthop. (2018) 89:652–5. 10.1080/17453674.2018.1519104 30326758 PMC6319184

[17] Shore BJ Graham HK Jr. Management of moderate to severe hip displacement in nonambulatory children with cerebral palsy. JBJS Rev. (2017) 5:e4. 10.2106/JBJS.RVW.17.00027 29256976

[18] Soo B Howard JJ Boyd RN Reid SM Lanigan A Wolfe R. Hip displacement in cerebral palsy. J Bone Joint Surg Am. (2006) 88:121–9. 10.2106/00004623-200601000-00015 16391257

[19] Connelly A Flett P Graham HK Oates J. Hip surveillance in Tasmanian children with cerebral palsy. J Paediatr Child Health. (2009) 45:437–43. 10.1111/j.1440-1754.2009.01534.x 19712179

[20] Horvath R Abicht A Holinski-Feder E Laner A Gempel K Prokisch H. Leigh syndrome caused by mutations in the flavoprotein (FP) subunit of succinate dehydrogenase (SDHA). J Neurol Neurosurg Psychiatry. (2006) 77:74–6. 10.1136/jnnp.2005.067041 16361598 PMC2117401

[21] Ghaoui R Sue CM. Movement disorders in mitochondrial disease. J Neurol. (2018) 265:1230–40. 10.1007/s00415-017-8722-6 29307008

[22] Sanger TD. Pathophysiology of pediatric movement disorders. J Child Neurol. (2003) 18(Suppl 1):S9–24. 10.1177/0883073803018001S0401 13677568

[23] Hagglund G Lauge-Pedersen H Wagner P. Characteristics of children with hip displacement in cerebral palsy. BMC Musculoskelet Disord. (2007) 8:101. 10.1186/1471-2474-8-101 17963501 PMC2194677

[24] Shrader MW Wimberly L Thompson R. Hip surveillance in children with cerebral palsy. J Am Acad Orthop Surg. (2019) 27:760–8. 10.5435/JAAOS-D-18-00184 30998565

[25] Shapiro F Zurakowski D Bui T Darras BT. Progression of spinal deformity in wheelchair-dependent patients with Duchenne muscular dystrophy who are not treated with steroids: coronal plane (scoliosis) and sagittal plane (kyphosis, lordosis) deformity. Bone Joint J. (2014) 96:100–5. 10.1302/0301-620X.96B1.32117 24395319

[26] Garg S Engelman G Yoshihara H McNair B Chang F. The relationship of gross motor functional classification scale level and hip dysplasia on the pattern and progression of scoliosis in children with cerebral palsy. Spine Deform. (2013) 1:266–71. 10.1016/j.jspd.2013.05.002 27927357

[27] Fujak A Raab W Schuh A Richter S Forst R Forst J. Natural course of scoliosis in proximal spinal muscular atrophy type II and IIIa: descriptive clinical study with retrospective data collection of 126 patients. BMC Musculoskelet Disord. (2013) 14:283. 10.1186/1471-2474-14-283 24093531 PMC3850509

[28] Senaran H Shah SA Glutting JJ Dabney KW Miller F. The associated effects of untreated unilateral hip dislocation in cerebral palsy scoliosis. J Pediatr Orthop. (2006) 26:769–72. 10.1097/01.bpo.0000242426.60995.29 17065943

[29] Lonstein JE Beck K. Hip dislocation and subluxation in cerebral palsy. J Pediatr Orthop. (1986) 6:521–6. 10.1097/01241398-198609000-00001 3760161

[30] Pritchett JW. The untreated unstable hip in severe cerebral palsy. Clin Orthop Relat Res. (1983) 173:169–72. 10.1097/00003086-198303000-00022 6825328

[31] Patel J Shapiro F. Simultaneous progression patterns of scoliosis, pelvic obliquity, and hip subluxation/dislocation in non-ambulatory neuromuscular patients: an approach to deformity documentation. J Child Orthop. (2015) 9:345–56. 10.1007/s11832-015-0683-7 26423268 PMC4619374

[32] Repko M Krbec M Chaloupka R Tichý V Sprláková-Puková A. Neuromuscular deformity of the pelvis and its surgical treatment. Acta Chir Orthop Traumatol Cech. (2008) 75:117. 18454916

[33] Frischhut B Krismer M Stoeckl B Landauer F Auckenthaler T. Pelvic tilt in neuromuscular disorders. J Pediatr Orthop B. (2000) 9:221–8. 10.1097/01202412-200010000-00003 11143463

[34] Hägglund G Pettersson K Czuba T Persson-Bunke M Rodby-Bousquet E. Incidence of scoliosis in cerebral palsy: a population-based study of 962 young individuals. Acta Orthop. (2018) 89:443–7. 10.1080/17453674.2018.1450091 29537343 PMC6600133

